# Supplementary material for: Effects of Heat Treatment on the Microstructure and Mechanical Properties of a Dual-Phase High-Entropy Alloy Fabricated via Laser Beam Power Bed Fusion
Source: Micromachines (Basel). 2024 Mar 29;15(4):471. doi: 10.3390/mi15040471 (PMC11052300; doi:10.3390/mi15040471)
Supplement: Supplementary file 1 [file micromachines-15-00471-s001.zip › micromachines-2884807-supplementary.pdf]

Effects of Heat Treatment on the Microstructure and Mechanical Properties of a Dual-Phase High-Entropy Alloy Fabricated via Laser Beam Power Bed Fusion

Xiaojun Tan [a,b,c,d], Zihong Wang [e], Haitao Chen [b], Xuyun Peng [b], Wei Zhang [c], Haibing Xiao [c,*], Zhongmin Liu [a,d], Yu Hu [f,g], Liang Guo [a,d,*], Qingmao Zhang [a,d]

[a] Guangdong Provincial Key Laboratory of Nanophotonic Functional Materials and Devices, School of Information and Optoelectronic Science and Engineering, South China Normal University, Guangzhou, 510006, China

[b] Sino-German Intelligent Manufacturing school, Shenzhen Institute of Technology, 518116, China

[c] Intelligent Manufacturing and Equipment School, Shenzhen Institute of Information Technology, Shenzhen, 518172, China

[d] Guangdong Provincial Key Laboratory of Industrial Ultrashort Pulse Laser Technology, Shenzhen, 518055, China

[e] College of Materials Science and Engineering, Chongqing University, Chongqing 400045, China

[f] School of Locomotive and Vehicle, Guangzhou Railway Polytechnic, Guangzhou 510430, China

[g] Guangdong-Hongkong-Macao Joint Laboratory of Energy Saving and Intelligent Maintenance for Modern Transportations, Guangzhou 510430, China

* Email: xiaohb@sziit.edu.cn, Guoliangchn@163.com

Supplementary Figure

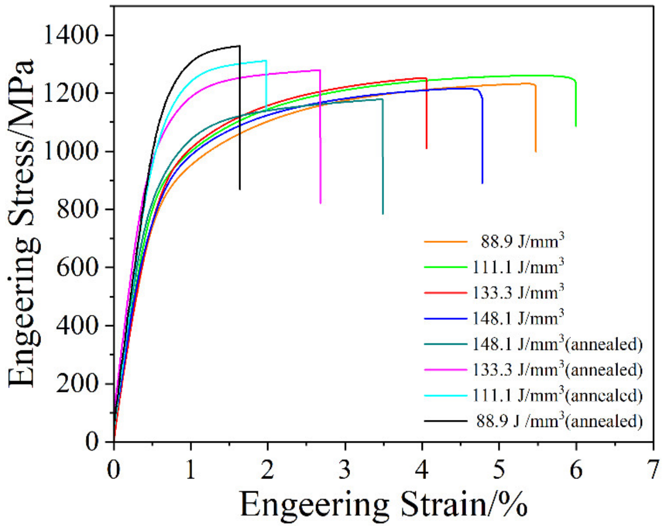

Figure S1 Tensile stress-strain curves at room temperature of as-printed and annealed HEA samples with different VEDs
